# Continuous thermodynamics approach for hydrogen solubility prediction

**DOI:** 10.1038/s41598-026-36303-w

**Published:** 2026-07-09

**Authors:** Wilfredo Angulo, María G. Lucena, Yris González, Dany De Cecchis, Alexander Espinoza, Juan P. Requez, Daniela Galatro

**Affiliations:** 1 School of Mathematical and Computational Sciences, Yachay Tech University, Urcuquí, 100115 Imbabura Ecuador; 2 Departamento de Ingeniería Química, Universidad Nacional Politécnica Antonio José de Sucre (UNEXPO), Barquisimeto, Venezuela; 3 Facultad de Ciencias Naturales y Matemáticas, Escuela Superior Politécnica del Litoral (ESPOL), Campus Gustavo Galindo Km. 30.5 vía Perimetral, Guayaquil, Ecuador; 4 Computational Science Research Center, San Diego State University, San Diego, CA USA; 5 Dirección de Investigación y Postgrado, Universidad Nacional Politécnica Antonio José de Sucre (UNEXPO), Barquisimeto, Venezuela; 6 Department of Chemical Engineering & Applied Chemistry, University of Toronto, 200 College Street, M5S 3E5 Toronto, ON Canada

**Keywords:** Continuous thermodynamics, vacuum gas oils, characterization, hydrogen solubility, Chemistry, Energy science and technology, Engineering, Mathematics and computing

## Abstract

Accurate characterization of heavy petroleum fractions remains challenging in hydroprocessing reactor modeling, where reliable hydrogen-solubility estimates in complex hydrocarbon mixtures are essential. Continuous thermodynamics has been successfully applied to represent crude oils and petroleum fractions in vapour–liquid equilibrium calculations through continuous composition distributions. Building on this framework, we propose a continuous-thermodynamics-based characterization methodology for vacuum gas oils and integrate it with the Augmented Grayson–Streed (AGS) approach to predict hydrogen solubility. Over temperature and pressure ranges of 459–653 K and 1.0–12.5 MPa, respectively, the proposed strategy reduces the global average absolute deviation with respect to experimental data from 22.5% to 11%. Beyond improving accuracy, the framework provides a systematic route to define a minimal set of pseudocomponents, reducing arbitrariness in heavy-fraction characterization while relying only on routinely measured laboratory data.

## Introduction

Hydrotreating and hydrocracking units in the oil industry can process various oil fractions, from naphtha to atmospheric residues, to generate products of high commercial value and with a lower concentration of contaminants^1–8^. In these units, the oil fraction is fed to the reactor in the presence of hydrogen and a catalyst, where it is partially vaporized according to the corresponding operating temperature and pressure^8^. The rate of the chemical reactions carried out in the reactor is a function of the equilibrium distribution coefficients, composition, and density of the liquid phase. Accurate reaction kinetics and liquid-vapor equilibrium are, therefore, required to model these reactors, to accurately predict the conversion, and, consequently, to estimate the optimal operating conditions at different feed conditions and/or catalysts^8,9^.

Developing and improving hydroprocessing reactor models rely on accurate hydrogen solubility values in the processed oil fractions. This thermodynamic property is complex to obtain experimentally for heavy fractions^10,11^. This is mainly due to the thermal instability of hydrocarbon mixtures at high temperatures and pressures, the inherent challenge of handling and injecting hydrogen at high pressures, and the complex analysis of the samples^11–13^. Hence, limited experimental data on vapor-liquid equilibrium (VLE) in oil and hydrogen fraction systems obtained under hydroprocessing conditions is available, particularly data associated with crude oils and residues^1,12^. Moreover, experimental studies are considered not an attractive option, mainly due to the risk associated with high pressure and temperature conditions, high cost, and time-consuming execution^4,14^.

On the other hand, predictive methods are a convenient and low-cost alternative to estimate hydrogen solubility in oil fractions at different hydroprocessing operational conditions, as they overcome the disadvantages of experiments^10,11^. However, prediction faces the challenge of achieving a correct characterization of heavy oil fractions, which are considered complex mixtures, since they are composed of several families of hydrocarbons with different molecular properties, such as kerosenes, olefins, aromatics, and naphthenes, in addition to heavy compounds such as resins and asphaltenes and impurities such as hydrogen sulfide^1–3,15^.

For more than six decades, general VLE principles have been used to develop methods for predicting hydrogen solubility in hydrocarbons, most of them for pure species, mixtures, and light petroleum fractions; however, these methods are limited in the determination of crude oils and heavy fractions^1,13^.

The methods for predicting hydrogen solubility in heavy oil fractions developed from the general principles of the VLE are based on the use of empirical or semi-empirical correlations, such as the equations of state (EOS)^10,16^. Among these are those proposed by Lal et al.^17^, who used the Peng-Robinson and Soave-Redlich-Kwong EOSs and the Grayson Streed (GS) method to estimate hydrogen solubility in Athabasca bitumen. Their EOS-based results were effectively contrasted with experimental data in a 50-308$^{\circ }$C temperature range and hydrogen partial pressures up to 24.8 MPa.

Riazi and Roomi^18^ proposed a method for estimating hydrogen solubility in pure hydrocarbons, petroleum fractions, and coal liquids, where the fugacity coefficient of hydrogen in the mixture was estimated using the Scatchard-Hildebrand theory. This method requires knowing the values of the boiling point and density of the solvent, but not their critical properties, which in most cases cannot be accurately estimated, while characterizing mixtures using the pseudocomponent method. However, in the case of heavy oil fractions, this method is not fully predictive since it requires the adjustment of the reduced fugacity of the considered hypothetically pure components.

Torres et al. ^7^ developed the Augmented Grayson Streed (AGS) method, which is based on the equilibrium of a pseudobinary system using the empirical correlations of the GS method and including the Flory entropic contribution term in the calculation of the activity coefficient. They determined the hydrogen solubilities in n-alkanes from n-C7 to n-C36 in heavy petroleum fractions and coal liquids. In the case of petroleum fractions, a simple and homogeneous pseudocomponent was considered, with average specific gravity and average molecular weight obtained from laboratory data. The rest of the physical properties required for estimating hydrogen solubility were then estimated. They reported the prediction in these mixtures with an average absolute deviation (AAD) of 30% compared to 55% estimated from the GS method, in the range of 80 to 380$^{\circ }$C and 6.3 to 258.9 bar.

Moreover, Saajanlehto et al.^3^ determined the hydrogen solubility in heavy oil fractions, measured with continuous flow equipment in a temperature range from 498 to 598 K and pressures from 2 to 11 MPa. Their results were compared with those of four predictive methods and by simulating four other heavy oil fractions taken from the literature. The selected methods were the Perturbed-Chain Statistical Association Fluid Theory (PC-SAFT) in combination with a pseudocomponent characterization for heavy crudes; the Peng-Robinson equation of state, together with a characterization of the mixtures according to the simple carbon number (SCN); the Shaw correlation^19^, based on the corresponding theory, and the method based on the Scatchard-Hildebrand theory^18^. The results obtained from this study show that, of the four methods compared, the one that estimates solubility most accurately is the PC-SAFT, with an overall AAD of 12%, at temperature and pressure conditions in the following ranges: 323-653 K and 0.3-10.0 MPa, respectively.

On the other hand, Aguilar-Cisneros et al.^20^ employed a procedure based on the Marrero-Gani (MG) group contribution method to characterize seven oil fractions. They used selected functional groups to assign a hypothetical chemical structure to the oil fraction through an optimization process in which the Gibbs free energy is minimized. The oil fraction was modeled using the thermodynamic method as if it were a pure component using the assigned molecular pseudostructure. They posed the VLE of 13 pure hydrocarbons and obtained solubility estimates below the experimental values in all cases. Subsequently, they estimated the hydrogen solubility in the characterized oil fractions over a temperature range from 323 to 623 K and pressure from 1.23 to 24.85 MPa. The results obtained with the method gave an AAD with respect to the experimental data in oil fractions between 6.82% and 17.93%.

Aguilar-Cisneros et al.^21^ also reviewed and modified the functional groups of the original research and included a new group in the characterization procedure of 12 heavy oil fractions, covering coal liquids, refinery products, and bitumen, improving the predictions with respect to the previous work while avoiding the adjustment of the model parameters. They employed two group contribution methods, the MG and the Joback-Reid (JR), and divided the oil fraction into different pseudocomponents. Hydrogen solubility was determined by solving the VLE using the Peng-Robinson EOS, combined with the UNIFAC solution model, through a modified Huron-Vidal mixing rule. The results show that the hydrogen solubility estimation improves slightly when the oil fraction is divided into three or six arbitrarily set pseudocomponents. They also stated that both group contribution methods (MG and JR) provide similar deviations, with a minimum overall AAD percentage of 15.1 and 16.9 for JR and MG, respectively.

In addition to predictive models derived from vapour–liquid equilibrium (VLE) principles, a growing number of studies have developed models based on machine learning techniques. These approaches are trained on experimental datasets to identify underlying patterns and, once trained, can be used to predict hydrogen solubility under conditions not explicitly included in the training data.

Safamirzaei and Modarress^22^ implemented an artificial neural network to estimate hydrogen solubility in several heavy n-alkanes, whereas Tatar et al.^23^ employed four different machine learning techniques-Decision Tree (DT), Random Forest (RF), Gradient Boosting (GB), and Extremely Randomized Trees (ET)-to predict hydrogen solubility in 15 n-alkanes (C$_1$–C$_{46}$) using 1845 experimental data points collected from 29 independent studies. Their analysis indicated that system pressure, dimensionless pressure, dimensionless temperature, and critical pressure exert the strongest influence on hydrogen solubility, in agreement with established thermodynamic models. Among the evaluated techniques, the RF model exhibited the best performance, with a regression coefficient ($R^2$) of 0.9755 and a root mean square error (RMSE) of 0.0050.

Gorji and Alopaeus^24^ applied a quantitative structure property relationship (QSPR) approach to predict hydrogen solubility in 32 pure hydrocarbons, using a dataset comprising 1751 experimental points over wide ranges of temperature and pressure. In addition, they employed a simple machine learning algorithm based on multilinear regression (MLR). The proposed MLR–QSPR models showed acceptable predictive capability for the validation dataset, with an average absolute deviation (AAD) of 9.79%.

Hadavimoghaddam et al.^25^ used two robust machine learning techniques-the group method of data handling (GMDH) and genetic programming (GP)-to develop advanced correlations for estimating hydrogen solubility in 32 pure hydrocarbons, including alkanes, alkenes, cycloalkanes, aromatics, polycyclic aromatics, and terpenes. Their database consisted of 1332 experimental measurements covering temperatures from 98 to 701 K and pressures from 0.101 to 78.45 MPa. The GP-based model outperformed the GMDH approach, yielding an $R^2$ value of 0.986 and an RMSE of 0.0132.

Nasery et al.^14^ applied an adaptive neuro fuzzy inference system (ANFIS) to estimate hydrogen solubility in four heavy petroleum fractions, achieving an AAD of 4.47%.^26^ implemented machine learning techniques to model hydrogen solubility in 11 cyclic and aromatic compounds, while^4^ employed five artificial intelligence models to predict hydrogen solubility in 26 pure hydrocarbons, demonstrating that the XGBoost model provides robust and reliable predictions with an AAD of 1.81%.

In a subsequent study,^11^ analyzed a dataset comprising 445 experimental measurements to estimate hydrogen solubility in fuels and various heavy petroleum fractions using four advanced machine learning models. Among them, XGBoost again showed the highest predictive accuracy, with an AAD of 1.41%.

Despite their strong predictive performance, the machine learning models reported in the aforementioned studies require large experimental datasets for training and have been developed predominantly for pure hydrocarbons. Consequently, their direct applicability to complex heavy petroleum fractions remains limited.

The referential framework previously described shows the scientific community’s interest in developing methods for determining hydrogen solubility in heavy oil fractions to improve the accuracy in predicting this thermodynamic property, which is essential to designing and operating hydroprocessing reactors. Additionally, different approaches for characterizing the heavy oil fraction, such as pseudocomponents, SCN, and the group contribution technique, are used. Furthermore, literature records another technique to characterize oil fractions: continuous thermodynamics. In this approach, the composition of the hydrocarbon mixture is described using a continuous distribution function, either analytically or numerically, with respect to one or more measurable variables, such as boiling point or molecular weight^27,28^. Their use can reduce mathematical complexity, allowing for eliminating randomness and arbitrariness in the determination of pseudocomponents^29^.

In this regard, Lucena et al.^5^ in a documentary review of scientific research on the applications of continuous thermodynamics in phase equilibrium calculations of complex mixtures, showed that this technique provides an effective alternative for the characterization of crude oils and various oil fractions, so it has great potential to be applied in the design and operation models of oil production and refining processes, especially in those where the VLE is established. Specifically, in determining hydrogen solubility in heavy oil fractions, the characterization technique plays a crucial role in the accuracy of the results. This thermodynamic variable is used to calculate the amount of hydrogen needed in hydrotreatment and hydrocracking processes, which varies according to the oil fraction processed. An excessive or deficient supply of hydrogen can lead to a waste of resources and energy, a potential safety risk in the plant, and a detriment to the quality of hydrotreated fuels^11,12^.

In this work, we propose a continuous-thermodynamics based methodology to characterize heavy oil fractions and to estimate hydrogen solubility in feedstocks typically processed in hydrotreating and hydrocracking reactors. The framework provides a systematic route to define a minimal set of pseudocomponents, reducing arbitrariness and improving the robustness of heavy fraction characterization. In addition, the proposed approach relies on limited laboratory information and is computationally inexpensive relative to the benchmark correlations considered and to data intensive machine learning and deep learning models.

## Experimental theoretical data

The heavy fractions of crude oil selected in this work are typical feed streams to hydroprocessing units, whose hydrogen solubility data was experimentally obtained, reported, and consequently published in scientific articles. Moreover, they have been used to evaluate the performance of predictive methods previously published in the literature. These streams include two gasoils from vacuum distillation columns: Athabasca light vacuum gasoil (LVGO) and Athabasca heavy vacuum gasoil (HVGO), and two residues from atmospheric distillation columns: Liaohe atmospheric residue (LHAR), and Venezuelan atmospheric residue (VNAR). Due to their thermo-physical properties these four oil fractions belong to the heaviest type of streams fed to hydroprocessing units in the oil industry^12,13,30^.

In this study, data on the physical properties of the heavy oil fractions analyzed here, as well as hydrogen-solubility data, were compiled from the literature on experimental methods and VLE-based correlations for hydrogen solubility in such mixtures.

A commercial process simulator was used to compute the thermophysical properties of the heavy oil fractions using laboratory inputs, namely density, molecular weight, and distillation curves. The input data are summarized in Table 1.

**Table 1:** Experimental physical properties of heavy petroleum fractions. Data for LVGO and HVGO were taken from^12^, and those for LHAR and VNAR from^13^ and^29^.

Property	LVGO	HVGO	LHAR	VNAR
Density @ 20°C (g/cm^3^)	0.892	0.973	0.9821	1.0297
Molecular weight (g/mol)	250	350	686.3	781.7
C (%)	85.0	84.4	86.83	83.82
H (%)	13.2	10.8	11.42	9.96
N (%)	0.4	1.5	0.36	4.31
S (%)	1.3	3.8	0.86	0.71
H/C ratio (mol/mol)	1.74	1.52	1.57	1.42
Distillation range ($^{\circ }$ C)	184–454	274–595	–	–
$\bullet$ IBP–350	–	–	1.8	1.5
$\bullet$ 350–420	–	–	52	3.5
$\bullet$ 420–500	–	–	24	19
$\bullet$ 500–FBP	–	–	69	76

However, for atmospheric residues (LHAR and VNAR), the distillation curve does not report the final boiling point (FBP). We therefore estimated the FBP, which is required to determine the mole fraction and molecular weight cutoffs. The FBP for LHAR and VNAR was obtained using a short algorithm that minimizes the root mean square error (RMSE) between the experimental average molecular weight, $MW_{\textrm{exp}}$, and the value predicted by the correlation of Liñan et al.^31^, $MW_{\textrm{cal}}$, which was developed specifically for atmospheric residues and is given by:

1 $$\begin{aligned} MW_{\textrm{cal}}=284\text{. }75\cdot [\exp (3\text{. }22\times 10^{-3}\cdot T_{\textrm{AVBP}})]\cdot [\exp (-2\text{. }52\cdot SG)]\cdot (T_{\textrm{AVBP}})^{8\text{. }3\times 10^{-2}}\cdot SG^{2\text{. }44} \end{aligned}$$

where *SG* is the specific gravity and $T_{\textrm{AVBP}}$ is the average boiling temperature (K). The latter is calculated from the distillation temperatures at 10 ($T_{10}$), 30 ($T_{30}$), 50 ($T_{50}$), 70 ($T_{70}$), and 90 ($T_{90}$) volume percent distilled, obtained from the ASTM D86 distillation curve, as follows:

2 $$\begin{aligned} T_{\textrm{AVBP}}=\dfrac{T_{10}+T_{30}+T_{50}+T_{70}+T_{90}}{5} \end{aligned}$$

The estimation procedure for the final boiling point is summarized in Algorithm 1.

**Algorithm 1:**
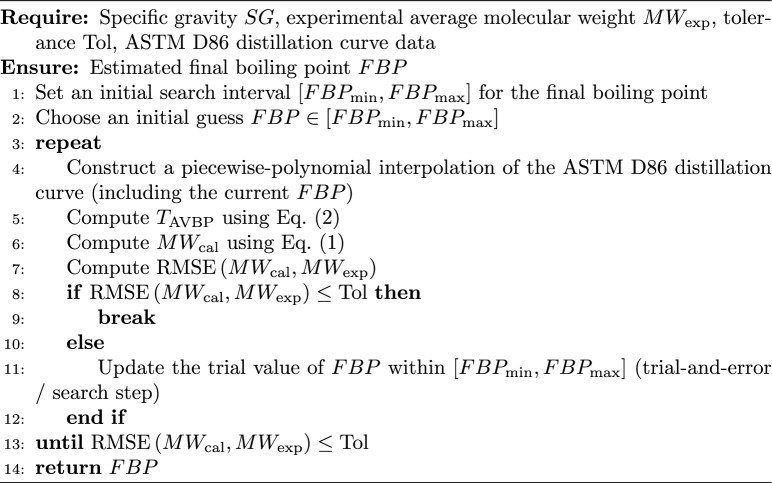
Estimation of the final boiling point (FBP) for atmospheric residues.

## Characterization through continuous thermodynamics

The characterization of the heavy fractions of crude oil is performed according to the general approach for implementing the continuous approach for VLE calculations described by Lucena et al.^5^ widely used in the literature^29,32,33^. This approach consists of the following steps:

1. selection of a continuous distribution function and an appropriate distribution variable to characterize the mixture;
2. estimation of the parameters of the selected distribution function;
3. calculation of the number, compositions, and cut points of the pseudocomponents using numerical quadrature; and
4. estimation of pseudocomponent physical properties from the quadrature points.

### Selected function and distribution variable

Typically, continuous thermodynamics applications in phase equilibrium calculations employ a single variable probability density function, which is sufficient to characterize a hydrocarbon mixture^5,34^. From a mathematical standpoint, this is the simplest case, since using more than one distribution variable would substantially increase the computational complexity. This simplicity not only makes the calculations more tractable but also enhances the feasibility of the method^35^.

After an exhaustive documentary review, the three-parameter gamma probability density function (PDF) was chosen to characterize heavy oil fractions. This PDF is given by:

3 $$\begin{aligned} F(P)=\dfrac{(P-\eta )^{\alpha -1}}{\beta ^{\alpha }\Gamma (\alpha )}\exp \biggl [-\dfrac{(P-\eta )}{\beta }\biggr ] \end{aligned}$$

where *P* is the distribution variable; *F*(*P*) is the probability density of *P*; $\alpha$ defines the form of the distribution; $\eta$ is the minimum value of *P* with a non-zero probability of ocurrence; and $\beta$ is a composite parameter defined in terms of $\alpha$, $\eta$ and the average value of *P*, $\bar{P}$, given by:

4 $$\begin{aligned} \bar{P}=\alpha \beta +\eta . \end{aligned}$$

Here, $\Gamma (\alpha )$ is the value of Euler’s Gamma Function at $\alpha$, which acts as a normalization constant to ensure that the total area under the distribution curve is exactly 1-an essential requirement for any probability density function.

This PDF has been successfully used to characterize a wide range of mixtures, including natural gas, middle distillates, crude oils, and various hydrocarbon mixtures^29,32,35,36^. Its practicality is further underscored by its successful application with three different distributed variables: molecular weight, boiling point, and number of carbon atoms, providing reliable performance in the characterization of medium and heavy crudes^5^. However, to our knowledge, it has not been applied to characterize vacuum gasoils and atmospheric residues, making its use in this study a pioneering effort in the field.

As for the distribution variable *P*, the molecular weight of the oil fraction is selected because this physical property is easily measurable in the laboratory. It is also the most used methodology for the characterization of oil fractions according to the continuous thermodynamics approach, which has allowed obtaining reliable results^6,34,36–40^. Accordingly, in this work *F*(*P*) represents the continuous molar distribution of components in the heavy oil fractions.

### Estimation of the parameters $\alpha$, $\beta$, and $\eta$

The continuous molar distributions *F*(*P*) of the heavy oil fractions were obtained by fitting the parameters $\alpha$, $\beta$, and $\eta$ of the PDF in Eq. (3) to the molecular weight data of the heavy oil cuts generated using the procedure described in Section 1. To this end, the algorithm proposed by Whitson^39,40^ was closely followed. However, instead of minimizing the sum of squared relative errors between the experimental molecular weight, $M_{i,\textrm{exp}}$, and the model predicted value, $M_{i,\textrm{model}}$, for the *i*th cut, the root mean square error , $\textrm{RMSE}_M(\alpha ,\beta ,\eta )$, was minimized, as defined by

5 $$\begin{aligned} \textrm{RMSE}_M(\alpha ,\beta ,\eta ) =\sqrt{ \frac{1}{(N-1)}\sum _{i=1}^{N-1} \left( M_{i,\textrm{exp}}-M_{i,\textrm{model}} \right) ^2} \end{aligned}$$

where *N* is the total number of cut-off points.

This metric provides a robust measure of model performance and indicates the quality of the fitted parameters $\alpha$, $\beta$, and $\eta$ in representing the molecular-weight distribution of the complex mixture^4^.

The estimation of the parameters $\alpha$, $\beta$, and $\eta$ can be posed as a constrained optimization problem in which the mismatch between the experimental and model predicted average molecular weights of the cuts is minimized^38^. Let $\{M_{i,\textrm{exp}}\}_{i=1}^{N-1}$ denote the experimental average molecular weights associated with the first $N-1$ cuts, and let $\{z_{i,\textrm{exp}}\}_{i=1}^{N-1}$ be the corresponding experimental mole fractions. For a given triplet $(\alpha ,\eta ,\beta )$, the model predicts the mole fraction of the *i*th cut through the cumulative distribution function (CDF)

6 $$\begin{aligned} \mathcal {F}(M;\alpha ,\beta ,\eta )=\int _{\eta }^{M}F(\xi ;\alpha ,\beta ,\eta )\,d\xi , \end{aligned}$$

so that: for $i=1,\ldots ,N-1$,

7 $$\begin{aligned} z_{i,\textrm{model}}(\alpha ,\beta ,\eta ,\underline{M}_i,\underline{M}_{i-1}) = \mathcal {F}(\underline{M}_i;\alpha ,\beta ,\eta ) - \mathcal {F}(\underline{M}_{i-1};\alpha ,\beta ,\eta ), \end{aligned}$$

where $\underline{M}_i$ denotes the (unknown) upper molecular weight bound that defines the *i*th cut, with $\underline{M}_0=\eta$.

In addition, the model predicted average molecular weight of the *i*th cut is obtained from the first partial moment of the distribution: for $i=1,\ldots ,N-1$,

8 $$\begin{aligned} M_{i,\textrm{model}}(\alpha ,\beta ,\eta ,\underline{M}_i,\underline{M}_{i-1}) =\eta +\frac{\alpha \beta \bigl [\mathcal {F}_1(\underline{M}_i;\alpha ,\beta ,\eta )-\mathcal {F}_1(\underline{M}_{i-1};\alpha ,\beta ,\eta )\bigr ]}{z_{i,\textrm{model}}(\alpha ,\beta ,\eta ,\underline{M}_i,\underline{M}_{i-1})}, \end{aligned}$$

where $\mathcal {F}_1(\cdot )$ denotes the corresponding CDF-like term required to compute the first moment (as in the Whitson formulation^40^), evaluated consistently with Eq. (6).

Under this formulation, the parameter estimation problem is stated as

9a $$\begin{aligned} \min _{\alpha ,\eta ,\{\underline{M}_i\}_{i=1}^{N-1}} \quad&\textrm{RMSE}_M(\alpha ,\eta ,\{\underline{M}_i\}) \end{aligned}$$

9b $$\begin{aligned} \text {s.t.}\quad&\beta =\frac{\bar{M}_{\textrm{exp}}-\eta }{\alpha } \end{aligned}$$

9c $$\begin{aligned}&\alpha _{\min }\le \alpha \le \alpha _{\max },\qquad \eta _{\min }\le \eta \le \eta _{\max } \end{aligned}$$

9d $$\begin{aligned}&0<\beta _{\min }\le \beta \le \beta _{\max } \end{aligned}$$

9e $$\begin{aligned}&z_{i,\textrm{model}}(\alpha ,\beta ,\eta ,\underline{M}_i,\underline{M}_{i-1}) = z_{i,\textrm{exp}}, \qquad i=1,\ldots ,N-1 \end{aligned}$$

9f $$\begin{aligned}&\underline{M}_{i-1}<\underline{M}_i,\qquad i=1,\ldots ,N-1 \end{aligned}$$

9g $$\begin{aligned}&\underline{M}_0=\eta ,\qquad \underline{M}_{N-1}<\underline{M}_N,\qquad \underline{M}_N\ \text {free (or sufficiently large)} \end{aligned}$$

where $\textrm{RMSE}_M(\alpha ,\eta ,\{\underline{M}_i\})$ refers to the following adaptation of (5)

10 $$\begin{aligned} \textrm{RMSE}_M(\alpha ,\eta ,\{\underline{M}_i\})=\sqrt{\frac{1}{N-1}\sum _{i=1}^{N-1}\Bigl (M_{i,\textrm{exp}}-M_{i,\textrm{model}}(\alpha ,\beta ,\eta ,\underline{M}_i,\underline{M}_{i-1})\Bigr )^2}, \end{aligned}$$

$\bar{M}_{\textrm{exp}}$ is the experimental overall average molecular weight, and $(\alpha _{\min },\alpha _{\max })$ and $(\eta _{\min },\eta _{\max })$ define admissible search intervals. Consistent with the constraint $\beta >0$, it is required that $\bar{M}_{\textrm{exp}}>\eta _{\max }$, and the bounds

11 $$\begin{aligned} \beta _{\min }=\frac{\bar{M}_{\textrm{exp}}-\eta _{\max }}{\alpha _{\max }},\qquad \beta _{\max }=\frac{\bar{M}_{\textrm{exp}}-\eta _{\min }}{\alpha _{\min }} \end{aligned}$$

ensure $\beta _{\min }\le \beta \le \beta _{\max }$ over the admissible domain.

In practice, the system of constraints in Eq. (9e) implicitly defines the cut bounds $\{\underline{M}_i\}$ for each candidate $(\alpha ,\eta )$ through a one dimensional trial and error (root-finding) procedure per cut, because each $\underline{M}_i$ must satisfy the mole fraction balance

12 $$\begin{aligned} \mathcal {F}(\underline{M}_i;\alpha ,\beta ,\eta )-\mathcal {F}(\underline{M}_{i-1};\alpha ,\beta ,\eta )=z_{i,\textrm{exp}}. \end{aligned}$$

Once the sequence $\{\underline{M}_i\}$ is obtained, the corresponding set of $\{M_{i,\textrm{model}}\}_{i=1}^{N-1}$ is computed from Eq. (8), and the objective function in Eq. (9a) is evaluated. The outer minimization with respect to $(\alpha ,\eta )$ (and, equivalently, $\beta$ via Eq. (9b)) can then be carried out with a nonlinear least squares solver (e.g., Levenberg–Marquardt), while the inner trial and error procedure in Eq. (12) enforces consistency with the experimental cut fractions.

### Number, composition, and cut points of the pseudocomponents

Once the continuous molar distribution *F*(*P*) has been determined, the distribution is discretized to obtain a finite, minimal set of pseudocomponents that adequately characterizes the heavy petroleum fraction^29,32–34,41–43^. This, in turn, enables the use of phase equilibrium calculation procedures originally developed for discrete components in mixtures whose composition is described by a continuous distribution^29,34,41^.

In this work, the discretization is performed using the quadrature method of moments (QMoM), which provides an optimal discrete representation of the continuous distribution while preserving its moments^34^. For the three parameter gamma probability density function adopted in this study (Eq. 3), the *k*th moment of the distribution is defined as

13 $$\begin{aligned} \lambda _k = \int _{\eta }^{\infty } P^{k} F(P)\, dP, \qquad k=0,1,\ldots \end{aligned}$$

where *P* denotes the molecular weight, $\alpha$, $\beta$, and $\eta$ are the fitted distribution parameters, and *F*(*P*) is the continuous molar distribution function.

To construct a quadrature approximation with *m* pseudocomponents, the first 2*m* moments $\{\lambda _k\}_{k=0}^{2m-1}$ of the distribution are computed analytically or numerically from Eq. (13). The QMoM then seeks a discrete representation of the form

14 $$\begin{aligned} F(P)\, dP \;\approx \; \sum _{j=1}^{m} w_j\, \delta (P-P_j), \end{aligned}$$

where $\delta (\cdot )$ is the Dirac delta function, $P_j$ are the quadrature abscissas, and $w_j$ are the corresponding quadrature weights.

The quadrature abscissas $\{P_j\}$ and weights $\{w_j\}$ are determined such that the discrete distribution exactly reproduces the first 2*m* moments of the continuous distribution, i.e.,

15 $$\begin{aligned} \sum _{j=1}^{m} w_j P_j^{k} = \lambda _k, \qquad k=0,1,\ldots ,2m-1. \end{aligned}$$

In the present work, this moment matching problem is solved using the *m*-point Gordon quadrature obtained through the product difference algorithm (PDA), which has been shown to provide higher accuracy and numerical stability than conventional Gaussian quadrature in the context of continuous thermodynamics^34,41^.

Within this framework, the number of pseudocomponents is directly given by the number of quadrature points *m*, the quadrature weights $w_j$ represent the molar fractions of the pseudocomponents, and the quadrature abscissas $P_j$ define the characteristic molecular weights (cut points) associated with each pseudocomponent.

The main steps of the discretization procedure are summarized in Algorithm 2.

**Algorithm 2:**
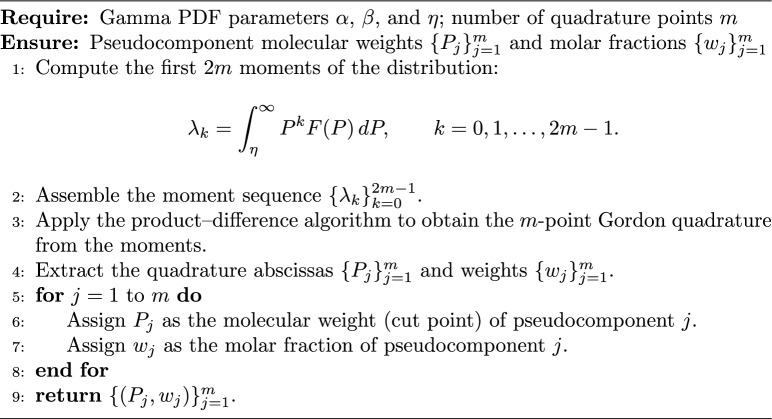
Discretization of the gamma molar distribution using the Quadrature Method of Moments (QMoM)

### Estimation of pseudocomponents physical properties

The heavy oil fraction is discretized into a finite, minimal set of pseudocomponents using the quadrature method of moments (QMoM). Each pseudocomponent is treated as a hypothetical pure component characterized by a representative molecular weight and a corresponding molar fraction. Accordingly, the molecular weight $MW_j$ and mole fraction $x_j$ of the *j*th pseudocomponent are directly obtained from the quadrature abscissas and weights, respectively.

The resulting set of pairs $(MW_j, x_j)$ constitutes the essential input for the estimation of the physical properties of both the pseudocomponents and the overall mixture. These properties are determined using empirical correlations widely reported in the scientific literature^44,45^, together with appropriate mixing rules.

The physical properties of the mixture are computed as mole fraction weighted averages of the corresponding pseudocomponent properties according to the following mixing rule:

16 $$\begin{aligned} \theta = \sum _{j=1}^{N} x_j\, \theta _j, \end{aligned}$$

where $\theta$ denotes a generic physical property of the mixture, $x_j$ is the mole fraction of the *j*th pseudocomponent, $\theta _j$ is the corresponding property of pseudocomponent *j*, and *N* is the total number of pseudocomponents used to represent the heavy oil fraction.

The specific correlations and methods employed to estimate the required physical properties of the pseudocomponents are summarized in Table 2.

**Table 2:** Physical property estimation methods adopted for the characterization of heavy petroleum fractions using discretized pseudocomponents.

Physical property	Estimation method
Critical pressure	Lee–Kesler correlation^45^
Critical temperature	Lee–Kesler correlation^45^
Acentric factor	Lee–Kesler correlation^45^
Molar volume	Definition based on critical properties^45^
Solubility parameter	SCN based approach^44^

## Hydrogen solubility estimation using the AGS approach

Hydrogen solubility is determined by solving the vapour–liquid equilibrium (VLE) problem for a multicomponent system consisting of hydrogen and the set of pseudocomponents representing the heavy oil fraction. The VLE condition is established by enforcing equality of fugacities of each component between the vapour and liquid phases at a given temperature and pressure. For component *i*, this condition can be expressed as

17 $$\begin{aligned} f_i^{V} = f_i^{L}. \end{aligned}$$

In this work, a heterogeneous thermodynamic formulation is adopted, in which the vapour phase fugacity coefficients are computed using a cubic equation of state, while non idealities in the liquid phase are accounted for through an activity coefficient model. Under this framework, the equilibrium relation can be written as

18 $$\begin{aligned} y_i \, \phi _i^{V} \, P = x_i \, \gamma _i \, f_i^{L*}, \end{aligned}$$

where $x_i$ and $y_i$ denote the mole fractions of component *i* in the liquid and vapour phases, respectively; $\phi _i^{V}$ is the vapour phase fugacity coefficient; $\gamma _i$ is the liquid phase activity coefficient; and $f_i^{L*}$ is the fugacity of the pure liquid component at system conditions.

The calculation of hydrogen solubility is performed using the Augmented Grayson–Streed (AGS) method^7^, which constitutes an extension of the classical Grayson–Streed model by incorporating an entropic correction based on Flory theory into the liquid phase activity coefficient. This modification improves the description of hydrogen solubility in heavy hydrocarbons by accounting for molecular size effects that become significant in systems involving very light solutes and heavy solvents.

Within the AGS framework, the liquid-phase activity coefficient of hydrogen at infinite dilution is expressed as the sum of an enthalpic contribution, described by regular solution theory, and an entropic contribution derived from Flory’s model. This combined formulation preserves the predictive character of the original Grayson–Streed approach while significantly improving its performance for heavy oil fractions.

The physical properties required by the AGS model for each pseudocomponent namely critical temperature, critical pressure, acentric factor, molar volume, and solubility parameter are those previously estimated from empirical correlations. The overall hydrogen solubility in the heavy oil fraction is obtained by solving the multicomponent VLE problem, yielding the hydrogen mole fraction in the liquid phase under specified temperature and pressure conditions.

The accuracy of the hydrogen solubility predictions is assessed by comparing the calculated liquid phase hydrogen mole fractions with experimental data available in the literature.

The prediction of hydrogen solubility for heavy oil fractions is performed under the same operational conditions used in the experimental determination, the average absolute deviation (AAD) is calculated, and the results of other solubility estimation methods published in the literature are compared^2,7,20,21^. The AAD percentage is selected as an indicator of the accuracy of the predictive methods, which consists of the average absolute deviation in percentage terms of the method results regarding the experimentally determined data at the laboratory level^21^. Particularizing this definition for hydrogen solubility in heavy oil fractions, expressed in terms of hydrogen mole fraction, the following expression:

19 $$\begin{aligned} \%\textrm{AAD} = \frac{100}{N_{d}} \sum _{i=1}^{N_{d}} \left| \frac{x_{H_2,\textrm{exp}}^{(i)} - x_{H_2,\textrm{cal}}^{(i)}}{x_{H_2,\textrm{exp}}^{(i)}} \right| , \end{aligned}$$

where $x_{H_2,\textrm{exp}}^{(i)}$ and $x_{H_2,\textrm{cal}}^{(i)}$ denote the experimental and calculated hydrogen mole fractions in the liquid phase, respectively, and $N_{d}$ is the total number of experimental data points considered.

Algorithm 3 summarizes the computational procedure used to estimate hydrogen solubility in heavy oil fractions. In this algorithm, the index *i* refers to experimental temperature–pressure conditions, *j* denotes pseudocomponents, and *k* represents the components involved in the vapour liquid equilibrium calculations.

**Algorithm 3:**
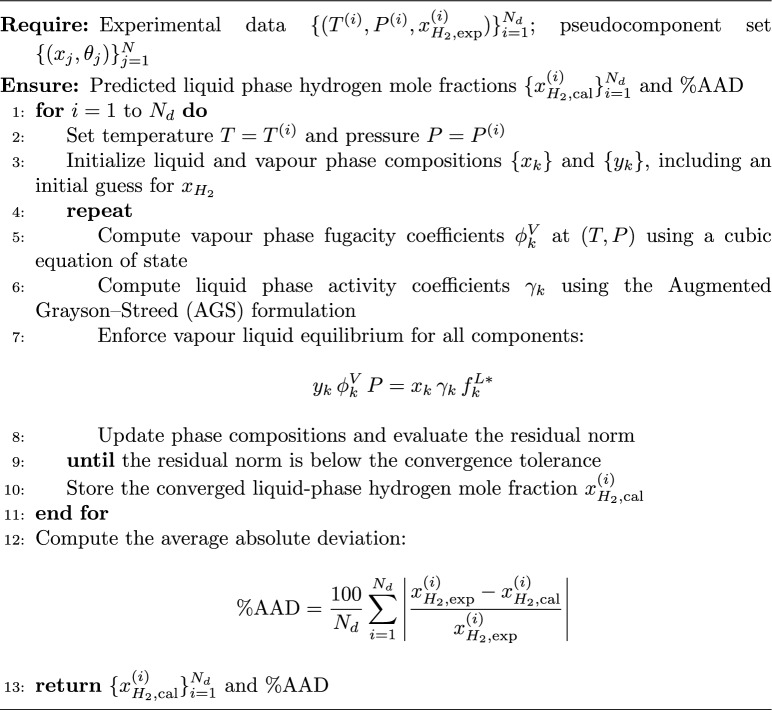
Hydrogen solubility estimation using the AGS method.

The number of pseudocomponents *N* is fixed by the QMoM discretization and is independent of the number of experimental data points $N_d$, which are used exclusively for model validation through the AAD.

## Results and discussion

Before presenting the results, it is worth noting that the numerical framework underlying this study was implemented using well established computational tools, with clearly defined optimization settings and convergence criteria. The constrained optimization problem associated with the estimation of the gamma distribution parameters was implemented in Python 3.11, taking advantage of its robust numerical optimization capabilities. The discretization of the continuous molar distribution using the quadrature method of moments (QMoM) was carried out in Wolfram Mathematica 13.2, leveraging its symbolic and numerical computation features. Subsequently, the estimation of pseudocomponent physical properties and the solution of the vapour–liquid equilibrium problem, including the AGS formulation, were performed in MATLAB R2018a using the fsolve routine to solve the resulting systems of nonlinear equations. Convergence tolerances of the order of $10^{-8}$ were imposed for all iterative procedures to ensure numerical stability and consistency, although results are reported using a limited number of significant digits for clarity and compact presentation. In addition, the sensitivity of the discretization with respect to the number of quadrature points was systematically analyzed, as discussed below, ensuring that the reported results are both numerically robust and reproducible.

### Continuous molar distribution of heavy fractions of crude oil

**Fig. 1:**
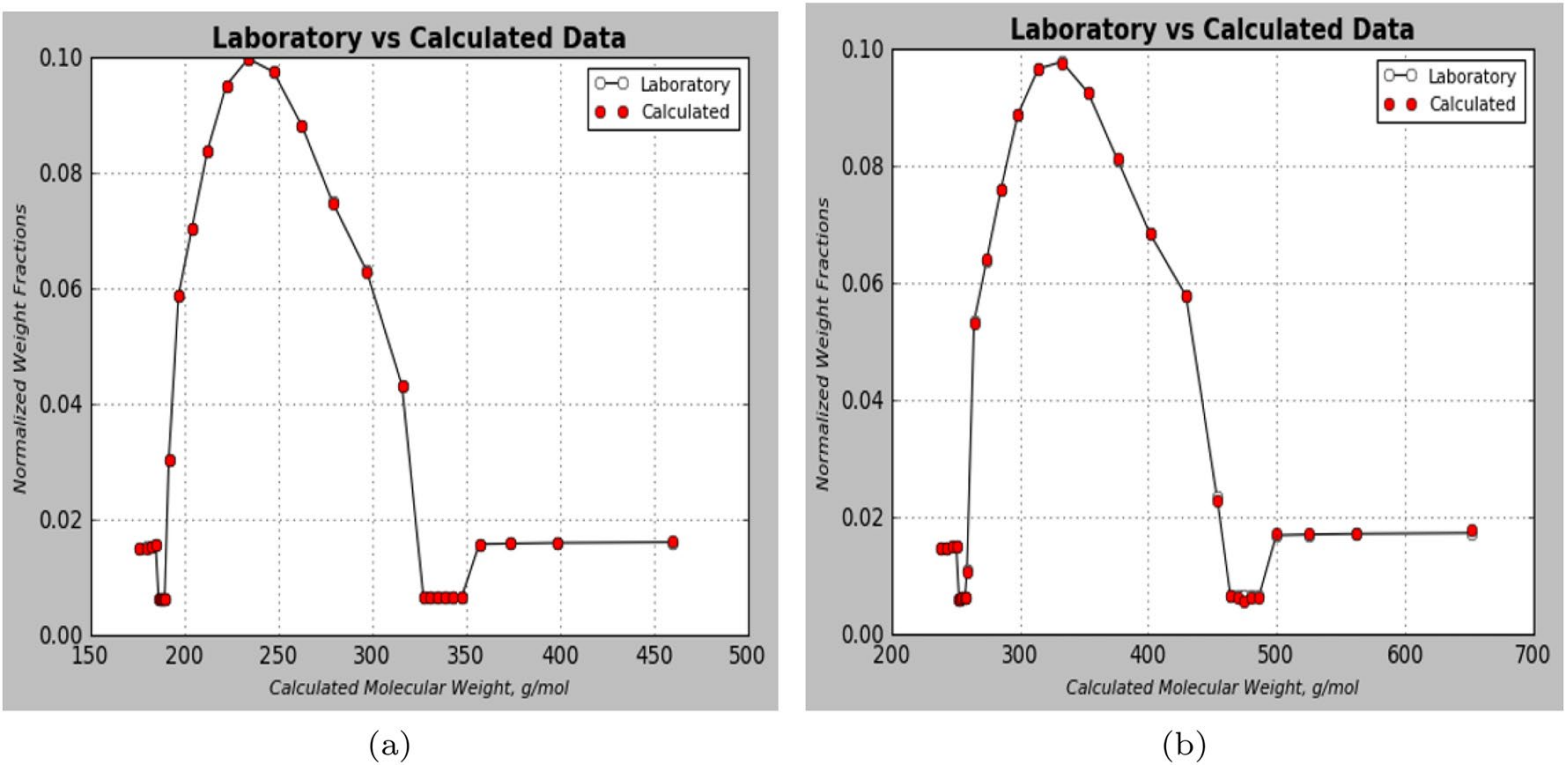
Continuous molar distribution (Laboratory vs Calculated Data): (**a**) for LVGO and (**b**) for HVGO.

The parameters of the gamma function representing the continuous molar distributions of the LVGO and HVGO vacuum gas oils are reported in Table 3. Figure 1 illustrates the excellent agreement between the experimental data and the distributions calculated using the selected probability density function. The RMSE values obtained from the constrained optimization used to estimate the parameters $\alpha$, $\beta$, and $\eta$ are close to zero, with values of 0.0033 and 0.0270 for LVGO and HVGO, respectively. These results demonstrate that the three-parameter PDF (3) is suitable for representing the continuous molecular-weight distribution not only of crude oils and light fractions, but also of vacuum gas oils^29,32,38,46,47^.

**Table 3:** Estimated parameters of the fitted three parameter gamma probability density function (PDF) for LVGO and HVGO, together with the corresponding RMSE.

Parameter values and RMSE	LVGO	HVGO
$\alpha$	1.7098	1.6679
$\beta$	38.9258	58.0185
$\eta$ (g/mol)	172.84	233.69
RMSE	0.0033	0.0270

The two molar distributions exhibit similar shapes, since both gas oils originate from the refining of the same Canadian crude oil. The estimated values of the shape parameter $\alpha$ are consistent with those reported in the literature, where reference values for reservoir fluids typically range from 0.5 to 2.5 and are generally lower than 25 for heavy crude oils^39,48^. The parameter $\eta$, which represents the molecular weight of the lightest compound in the hydrocarbon mixture, is lower for LVGO (Fig. 1a), as expected for the lighter fraction. Similarly, the parameter $\beta$, which controls the positive skewness of the distribution by stretching the right-hand tail, attains a higher value for HVGO (Fig. 1b), reflecting its higher average molecular weight.

**Fig. 2:**
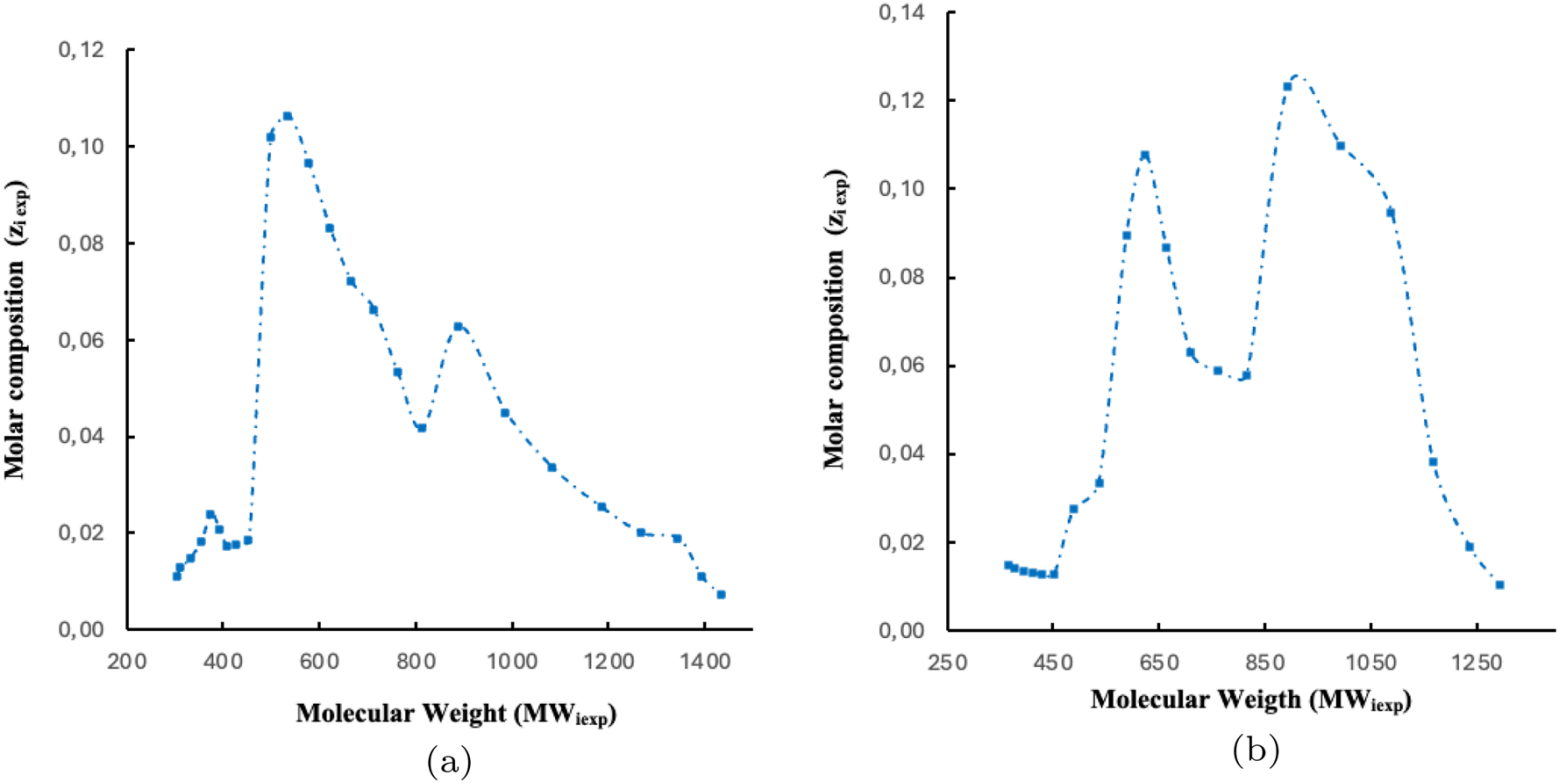
Theoretical experimental data for the heavy petroleum fraction: (**a**) for LHAR and (**b**) for VNAR.

In contrast, for the LHAR and VNAR atmospheric residues, the experimental data cannot be adequately represented by the selected gamma function. In both cases, the molecular weight distributions exhibit a bimodal behavior, as shown in Fig. 2a,b.

Atmospheric residues are the heaviest fractions obtained from atmospheric distillation columns during crude oil refining. They contain hydrocarbons with carbon numbers typically greater than 25, high sulfur contents, and trace metals such as nickel and vanadium. These fractions are characterized by the presence of asphaltenes, which are complex organic compounds composed of polyaromatic structures linked by saturated chains^8,45^. As a result, atmospheric residues constitute more complex hydrocarbon mixtures than vacuum gas oils, as evidenced by the bimodal nature of their molecular weight distributions. This behavior indicates the presence of more than one dominant molecular weight population, associated with different distillation cuts, and explains why a unimodal three parameter gamma PDF cannot adequately represent these fractions.

In this regard, Cotterman et al.^35^ pointed out that the characterization of complex hydrocarbon mixtures in VLE calculations may require the use of composite distributions formed by the sum of two unimodal functions. However, no practical methodology for implementing such an approach was developed in their work. Similarly, Lucena et al.^5^, in a comprehensive review of continuous thermodynamics applications to phase equilibrium calculations, concluded that further research is needed on the characterization of hydrocarbon mixtures using bimodal or multimodal distribution functions.

Based on the observed molecular weight distributions of atmospheric residues, these fractions should be characterized using a bimodal PDF, together with a suitable parameter estimation methodology requiring minimal experimental input. The development of such an approach is beyond the scope of the present work and is therefore proposed as a topic for future research.

### Discretized pseudocomponents of vacuum gas oils

In order to determine the physical properties of vacuum gas oils and achieve a complete characterization of these fractions, they are represented as mixtures of pseudocomponents obtained by discretizing the continuous molar distribution. The molecular weights and mole fractions of these pseudocomponents are computed using the Quadrature Method of Moments (QMoM), as described in the methodology section.

The quality of the discretization is assessed by evaluating the root mean square error (RMSE) between the continuous molar distribution function reconstructed from its fitted parameters $(\alpha ,\beta ,\eta )$ and its discretized approximation obtained through a Legendre polynomial expansion, whose roots correspond to the quadrature points determined by the product difference algorithm (PDA)^34^. Table 4 reports the RMSE values obtained for different numbers of quadrature points used in the discretization of the LVGO and HVGO molar distributions. In both cases, the RMSE decreases monotonically as the number of quadrature points increases, indicating a progressive improvement in the accuracy of the discretized representation. A significant reduction in RMSE is observed when increasing the number of quadrature points from $m=6$ to $m=8$, while further refinement beyond this value leads to more moderate improvements. This behavior confirms that a relatively small number of quadrature points is sufficient to accurately capture the main features of the continuous molar distribution, highlighting the efficiency of QMoM in generating a minimal yet representative set of pseudocomponents for heavy oil fractions.

**Table 4:** RMSE values obtained for different numbers of quadrature points *m* in the discretization of the LVGO and HVGO molar distributions.

LVGO		HVGO	
Quadrature points *m*	RMSE	Quadrature points *m*	RMSE
6	0.004350	6	0.003836
8	0.001725	8	0.001631
12	0.000737	12	0.000728

Based on these results, a value of $m=12$ quadrature points was selected for the discretization of the LVGO and HVGO molar distributions in all subsequent calculations. Although the improvement in RMSE from $m=8$ to $m=12$ is less pronounced than that observed for lower values of *m*, the additional quadrature points provide a more accurate representation of the distribution tails while maintaining a reasonable computational cost^34,41^. This choice minimizes numerical errors that could propagate into the estimation of pseudocomponent properties and subsequent vapour–liquid equilibrium and hydrogen solubility calculations.

**Fig. 3:**
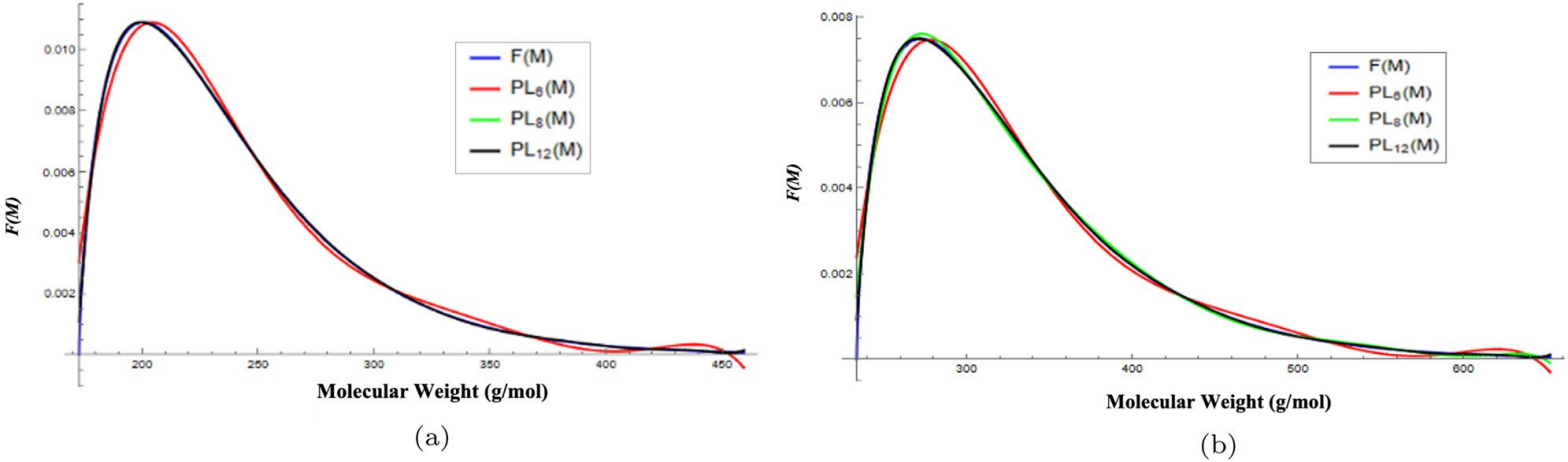
Legendre polynomial approximations of the molar distribution of vacuum gas oils: (**a**) LVGO and (**b**) HVGO.

The suitability of selecting $m=12$ is further illustrated in Fig. 3, which compares the continuous gamma distribution function *F*(*M*) with its discretized, piecewise linear reconstructions obtained using $m=8$ and $m=12$ quadrature points for LVGO and HVGO. In both cases, the discretization with $m=12$ closely reproduces the location and magnitude of the distribution peak, as well as the behavior in the heavy molecular weight tail. In contrast, the approximation obtained with $m=8$ exhibits small but noticeable deviations in regions of rapidly varying slope and at higher molecular weights.

The final set of pseudocomponents obtained using $m=12$ quadrature points is summarized in Table 5, which reports the quadrature abscissas (representative molecular weights $MW_i$) and the corresponding weights ($z_i$) for both LVGO and HVGO.

**Table 5:** Quadrature abscissas (molecular-weight points $MW_i$) and associated weights ($z_i$) obtained with $m=12$ for the LVGO and HVGO fractions.

*m*	LVGO		HVGO	
	$z_i$	$MW_i$	$z_i$	$MW_i$
1	0.03614	176.753	0.03845	239.313
2	0.13073	187.164	0.13465	254.575
3	0.21263	203.774	0.21420	279.007
4	0.22520	226.136	0.22352	311.900
5	0.17715	253.569	0.17431	352.202
6	0.11132	285.102	0.10921	398.439
7	0.05902	319.396	0.05803	448.613
8	0.02774	354.679	0.02746	500.110
9	0.01213	388.704	0.01213	549.650
10	0.00513	418.808	0.00519	593.378
11	0.00210	442.143	0.00215	627.204
12	0.00069	456.127	0.00071	647.446

**Fig. 4:**
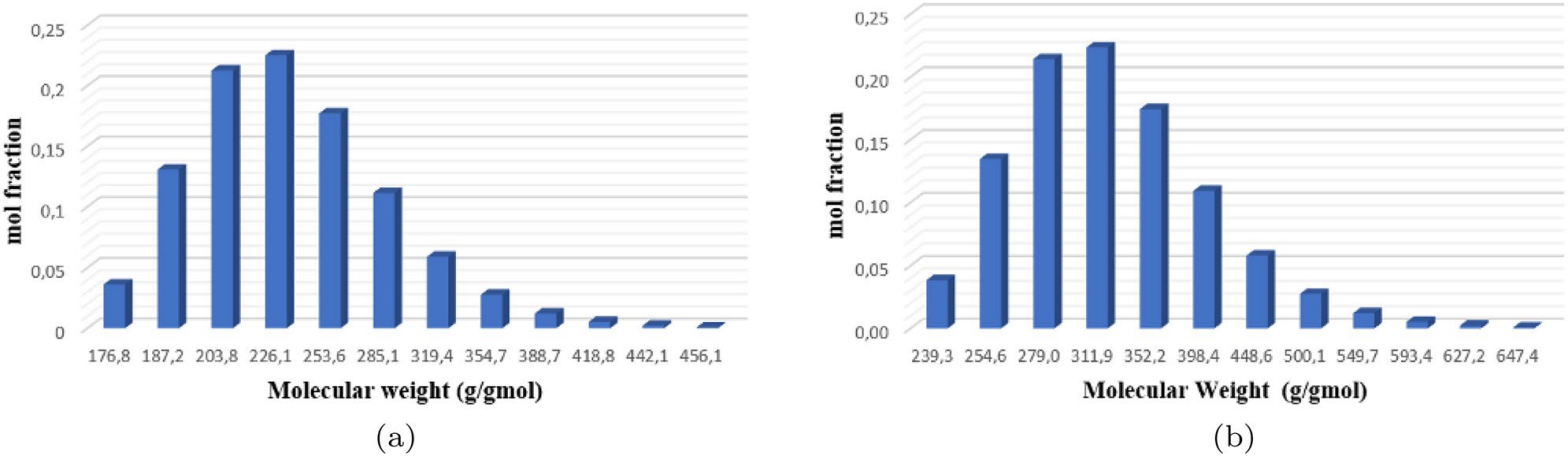
Discretized pseudocomponents of the continuous molar distribution: (**a**) LVGO and (**b**) HVGO.

The resulting pseudocomponent distributions span the entire molecular weight range of each fraction, with higher weights concentrated around the modal region of the distribution and progressively smaller weights assigned to heavier components. This behavior is illustrated in Fig. 4a,b.

This distribution of pseudocomponents reflects the underlying continuous molar distribution and ensures that both the dominant molecular weight populations and the heavy tail behavior are adequately captured. Consequently, the pseudocomponent sets reported in Table 5 provide a physically meaningful and numerically robust basis for the estimation of thermophysical properties and for subsequent vapour–liquid equilibrium and hydrogen solubility calculations.

### Prediction of hydrogen solubility of vacuum gasoils

The AGS^7^ method is implemented to evaluate the characterization methodology of vacuum gas oils under the continuous thermodynamics approach in the prediction of hydrogen solubility, which considers that these mixtures are a simple and homogeneous pseudocomponent with specific gravity and average molecular weight known from laboratory data, with which they determine the rest of the physical properties. That is, they do not perform a detailed characterization of the oil fractions used in their research, which is considered as an opportunity for improvement of the method.

**Fig. 5:**
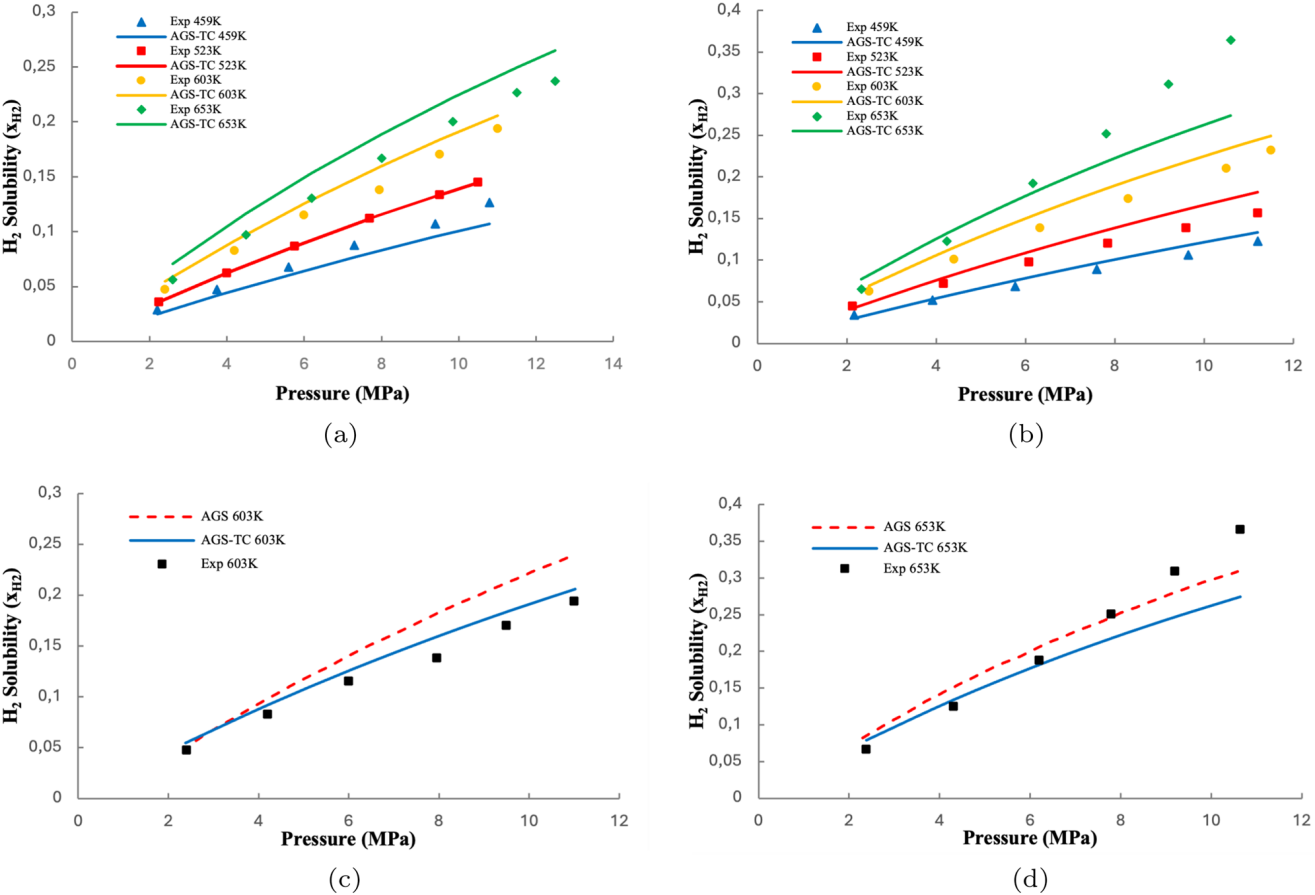
Prediction of hydrogen solubility in vacuum gas oils: (**a**) LVGO at 459 K, 523 K, 603 K and 653 K. (**b**) HVGO at 459 K, 523 K, 603 K and 653 K. (**c**) Comparison in prediction of hydrogen solubility in LVGO at 603 K, and (**d**) comparison in prediction of hydrogen solubility in HVGO at 653 K.

From the physical properties estimated for both vacuum gas oils, hydrogen solubility in these oil fractions is determined in a temperature range of 459-653 K and pressures of 1.0-12.5 (Table 6). The solubility increases with increasing pressure and temperature (Fig. 5), which is the expected behavior according to the experimental measurements of this property.

**Table 6:** Hydrogen solubility prediction using the AGS method and characterization under the continuous thermodynamics approach for LVGO and HVGO.

Fraction	*T*-range (°C)	*P*-range (MPa)	$\mathrm {H_{2}}$ solubility range (% mol)	AAD (%)
LVGO	186–380	2.2–12.5	2.5–26.5	10.3
HVGO	186–380	1.0–11.5	2.9–27.3	11.6

In the case of LVGO (Fig. 5a), the AGS method combined with the characterization under the continuous thermodynamics approach (AGS-CT) underestimates the hydrogen solubility at temperatures below 500 K, regarding the experimental data at the same conditions. The opposite is true for temperatures above 600 K, predicting higher values than the experimental ones. This deviation, with an average absolute deviation in the indicated temperature and pressure ranges of 10.3%, has significant implications for the accuracy of the AGS-CT method and the understanding of hydrogen solubility.

For HVGO, the AGS-CT overestimates the solubility for temperatures between 459 and 603 K. However, for the highest temperature of 653 K the values are below the experimental values for pressures from 6 to 11.5 MPa (Fig. 5b). An average absolute deviation in the indicated temperature and pressure ranges of 11.6% is obtained.

With the hydrogen solubility results reported by AGS7, for LVGO at 603 K, the results obtained for LVGO at 603 K are compared for the same range of pressures. As shown in Fig. 5c, although the two methods overestimate the value of this property, the AGS-CT does it more accurately. For HGVO, the solubility results are compared at 653 K; in this case the improvement in the AGS-CT results can only be evident for pressures between 2 and 6 MPa, since above this value the AGS reports results closer to the experimental values (Fig. 5d). However, in terms of the percentage AAD of the estimated results compared with the experimental data, the AGS-CT manages to reduce the AAD for LVGO from 14% to 10.3% and for HVGO from 31% to 11.6% and the overall AAD from 22.5% to 11%.

The results obtained for two vacuum gas oils, with typical properties of the feed to the hydroprocessing units, suggest that the characterization methodology implemented under the continuous thermodynamics approach and starting from minimum laboratory data improves the prediction of hydrogen solubility in these fractions.

Table 7 compares the average absolute deviation (AAD) of the predicted hydrogen solubility in LVGO and HVGO obtained with several vapour–liquid equilibrium (VLE) based methods reported in the literature, namely: the original Augmented Grayson–Streed (AGS) model^7^; the PC-SAFT approach reported by Saajanlehto et al.^3^; the modified group contribution method MG1 (Mod. MG1) reported by Riazi and Roomi^18^; and the modified group contribution methods MG2 (Mod. MG2) and JR (Mod. JR) reported by Aguilar-Cisneros et al.^21^. The table also includes the results of the proposed AGS–CT approach, which combines the AGS framework with the continuous-thermodynamics-based characterization introduced in this work. For each fraction and method, the corresponding temperature and pressure ranges of the experimental dataset used for validation are reported together with the resulting %AAD.

**Table 7:** Comparison of average absolute deviation (AAD) values for hydrogen solubility prediction of LVGO and HVGO using different predictive methods.

		**Predictive methods**					
Fraction	Variables	AGS	PC-SAFT	Mod. MG1	Mod. MG2	Mod. JR	AGS-CT
LVGO	Temp. (K)	353–653	353–653	298–448	353–603	353–603	459–653
	Press. (MPa)	1.17–12.27	1.0–10.0	1.94–12.28	1.97–11.53	1.97–11.53	2.0–11.0
	%AAD	14	10	12.4	13.1	16.7	10.3
HVGO	Temp. (K)	353–653	353–523	283–448	353–653	353–653	459–653
	Press. (MPa)	0.63–11.88	1.0–10.0	1.97–11.53	1.97–12.27	1.97–12.27	1.0–11.5
	%AAD	31	19	13.8	11.8	10.4	11.6

As mentioned above, the characterization under the continuous thermodynamics approach manages to decrease the deviation obtained by means of the AGS method^7^. In the specific case of LVGO when compared with the rest of the AADs, only that of the method of Saajanlehto et al.^3^ turns out to be lower. However, it is lower by only 1%.

Regarding the AAD of hydrogen solubility in HVGO obtained with the different methods, the AGS-CT manages to reduce the deviation obtained by the AGS method^7^; in this case, it is reduced by 62%, which is considered a significant decrease. Compared with the rest of the AADs, only that of the Aguilar-Cisneros et al.^21^ method is lower, specifically that estimated with the JR model; however, the difference is only 1.2%.

Even though the scientific literature has published results of deviations in the same order of magnitude as those achieved with the AGS-CT, it is confirmed that the characterization under the continuous thermodynamics approach contributes to the improvement in the prediction of hydrogen solubility of vacuum gas oils using the AGS method and additionally achieves AAD values lower than those of the other three predictive methods for this type of mixtures.

It should be noted that the remaining systematic deviations observed in the hydrogen-solubility predictions such as slight underprediction at lower temperatures and overprediction at higher temperatures are not solely attributable to characterization uncertainties. These trends rather reflect intrinsic limitations of the Augmented Grayson–Streed (AGS) model itself. The AGS formulation relies on semi-empirical assumptions derived from regular solution theory combined with an entropic correction based on Flory theory, whose validity may deteriorate outside specific temperature and pressure ranges^7^. In particular, the temperature dependence of hydrogen heavy hydrocarbon interactions and the simplified treatment of excess Gibbs energy can lead to systematic biases under extreme conditions^49,50^. While the improved characterization strategy proposed in this work significantly reduces uncertainty associated with pseudocomponent definition, it does not alter the fundamental thermodynamic structure of the AGS model. Therefore, the observed deviations highlight the inherent predictive limits of the AGS approach rather than deficiencies in the proposed continuous-thermodynamics-based characterization.

The results of this research are a valuable scientific contribution to improving the models designed for monitoring and operating the hydroprocessing reactors since they contribute to determining with greater precision the amount of hydrogen required. This represents a potential increase in productivity in the hydrotreating and hydrocracking units because it avoids unnecessary hydrogen consumption in the reactors; excessive hydrogen content in the final product, which reduces the quality of the products generated; additional energy consumption and safety risks associated with the increase in pressure due to hydrogen handling in the equipment.

The above is related to the increase in productivity, since it is a contribution to the efficient use of hydrogen, a high-cost raw material required in hydroprocessing units for the elimination of unwanted impurities in petroleum fractions, such as sulfur, nitrogen, oxygen, etc.; to improve the quality of various fuels such as diesel and gasoline, and also to reduce emissions of polluting gases (SOx and NOx) during their consumption.

#### Remark 1

It should be emphasized that the objective of the proposed methodology is not to outperform all existing thermodynamic or equation of state based models in terms of absolute predictive accuracy. Rather, its main contribution lies in providing a systematic, transparent, and low-data characterization framework for heavy oil fractions within the continuous thermodynamics formalism. While advanced models such as PC-SAFT or group contribution based approaches may achieve comparable or, in some cases, slightly lower deviations, they typically require extensive parameterization, detailed compositional information, or higher computational effort. In contrast, the proposed approach achieves comparable accuracy using limited laboratory data and a minimal, well defined set of pseudocomponents, making it particularly attractive for engineering applications where data availability and computational efficiency are critical.

## Conclusions

The characterization methodology developed in this work for two vacuum gas oils under the continuous thermodynamics framework, using laboratory data such as molecular weight, specific gravity, and distillation curves, leads to a significant reduction in the deviations of hydrogen solubility predicted by the Augmented Grayson–Streed (AGS) method^7^. In particular, the average absolute deviation (AAD) for LVGO is reduced from 14% (reported using the original AGS approach) to 10.3%, while for HVGO it decreases from 31% to 11.6%, over a wide and industrially relevant range of temperatures (459–653 K) and pressures (1.0–12.5 MPa).

Reliable estimation of hydrogen solubility in vacuum gas oils commonly used as feedstocks in hydroprocessing units contributes to a more accurate determination of hydrogen demand in reactors. This, in turn, helps to avoid excessive hydrogen consumption, which negatively impacts both material and energy costs as well as the quality of the hydrotreated fuels.

The continuous characterization of LVGO and HVGO is achieved by fitting a three parameter gamma probability density function to the experimental theoretical molecular weight data. The distribution parameters are estimated using a modified version of the Whitson algorithm^39,40^, in which the objective function is reformulated, resulting in an excellent fit and high reliability of the calculated distributions. While this unimodal PDF proves effective for representing vacuum gas oils, it is not suitable for atmospheric residues, whose molecular-weight distributions exhibit multimodal behavior.

The physical properties required for hydrogen solubility estimation are determined using well-established empirical correlations, treating the vacuum gas oils as mixtures of discretized pseudocomponents. These pseudocomponents are obtained via the quadrature method of moments (QMoM)^34^, yielding a discrete representation composed of 12 pseudocomponents for each fraction. The resulting RMSE values tend toward zero, confirming that the molecular weights and mole fractions obtained through this discretization are reliable and numerically robust.

Overall, the main strength of the proposed methodology lies in its systematic, low data, and computationally efficient characterization of heavy oil fractions within a continuous thermodynamics framework, achieving predictive accuracy comparable to advanced equation-of-state and group-contribution methods. Its limitations are primarily associated with the intrinsic assumptions of the AGS model and the use of unimodal distribution functions, which may restrict applicability under extreme conditions or for highly complex fractions such as atmospheric residues. These aspects define clear directions for future research.

## Data Availability

The datasets analyzed during the current study are taken from previously published sources, as detailed in the manuscript. No new datasets were generated during the current study.
